# Lithium Niobate Micromachining for the Fabrication of Microfluidic Droplet Generators

**DOI:** 10.3390/mi8060185

**Published:** 2017-06-09

**Authors:** Giacomo Bettella, Gianluca Pozza, Sebastian Kroesen, Riccardo Zamboni, Enrico Baggio, Carlo Montevecchi, Annamaria Zaltron, Ludovic Gauthier-Manuel, Giampaolo Mistura, Claudio Furlan, Mathieu Chauvet, Cornelia Denz, Cinzia Sada

**Affiliations:** 1 Physics and Astronomy Department, University of Padova, Via Marzolo 8, 35131 Padova, Italy; giacomo.bettella@unipd.it (G.B.); gianluca.pozza@unipd.it (G.P.); riccardo.zamboni@phd.unipd.it (R.Z.); enricobaggio90@gmail.com (E.B.); carlo.montevecchi@gmail.com (C.M.); annamaria.zaltron@unipd.it (A.Z.); giampaolo.mistura@unipd.it (G.M.); 2 Nonlinear Photonics Group, Institute of Applied Physics, University of Münster Corrensstrasse 2/4, 48149 Münster, Germany; Sebastian.Kroesen@de.TRUMPF.com (S.K.); denz@uni-muenster.de (C.D.); 3 FEMTO-ST Institute, UMR 6174, University of Bourgogne Franche-Comté, 15B Avenue des Montboucons, 25000 Besançon, France; ludovic.gauthier-manuel@univ-fcomte.fr (L.G.-M.); mathieu.chauvet@univ-fcomte.fr (M.C.); 4 CEASC at University of Padova, Via Jappelli 1, 35131 Padova, Italy; claudio.furlan@unipd.it

**Keywords:** microfluidics, droplets, lithium niobate, micromachining, laser ablation, femtosecond, dicing

## Abstract

In this paper, we present the first microfluidic junctions for droplet generation directly engraved on lithium niobate crystals by micromachining techniques, preparatory to a fully integrated opto-microfluidics lab-on-chip system. In particular, laser ablation technique and the mechanical micromachining technique are exploited to realise microfluidic channels in T- and cross junction configurations. The quality of both lateral and bottom surfaces of the channels are therefore compared together with a detailed study of their roughness measured by means of atomic force microscopy in order to evaluate the final performance achievable in an optofluidic device. Finally, the microfluidics performances of these water-in-oil droplets generators are investigated depending on these micromachining techniques, with particular focus on a wide range of droplet generation rates.

## 1. Introduction

Microfluidics was originally developed in 70 s for ink-jet printing, but in the last several decades, it has found a wide application in several research fields, especially in biotechnology [1,2,3] and micro-analytical chemistry [4]. As a matter of fact, thanks to microfluidic technology, reagents can be strongly reduced from millilitres and microliters to nanolitres and femtolitres, whereas hours of reaction time can be decreased to mere seconds or less [5].

Due to the strong advances in fluid handling techniques at the micron-scale, sophisticated microfluidic networks were realised, including all kinds of devices (like pumps, valves, fluid mixers and microchannels) to manipulate, deliver and control fluids within one compact chip [6,7].

On this basis, the lab-on-a-chip technology has gained broad success, by allowing several biological analyses and diagnoses to be conducted in parallel onto portable and miniaturized chips based on planar micro-fluidic platforms. Although microfluidics was born from silicon transferring micro-fabrication techniques from electronics, nowadays, the most employed materials for the realisation of microfluidic devices are polymers. Thermal reticulating polymers like polydimethylsiloxane (PDMS) and polyethylmethacrylate (PMMA), or light curing polymers like SU8, are widely used since standard moulding and photolithographic techniques respectively allow for obtaining any two-dimensional patterns needed for microfluidics applications [8,9].

Since polymeric devices are very cheap and fabrication processes have a good reproducibility, they are still dominating the market on disposable chips [10,11,12].

When durable multitasking integrated chips able to provide more sophisticated analyses and samples manipulation are needed, other materials seem to be more efficient. In fact, these devices often require optical and electronic stages, which cannot be realised on polymers because they are too expensive to rely on their poor durability. This is surely the case when more durable, chemical resistant and biological compatible materials are required [13,14], and inorganic based materials are strongly preferred.

A successful example is represented by the chemical etching and mechanical micromachining of silicon inherited from microelectronics [15,16]. Many works were published on the exploitation of laser micromachining as well, applied to different materials such as glass, silicon and ceramics, as recently reviewed in [17] and references therein quoted. These fabrication techniques were exploited also for realising waveguides and ring-resonators for sensing optical stages [18], demonstrating their flexibility and high performances.

However, the same micromachining technique was rarely used for integrating both integrated optics circuitry and microfludics on the same substrate: pioneering works on this was reported on silica by [19] using femto laser ablation techniques. In this scenario, quite surprisingly, lithium niobate (LN) has been rarely considered as a substrate to embed microfluidic circuitry, although it could be a valid alternative to glass and silicon for the integration of multiple stages able to produce, manipulate, sort droplets and analyse their contents.

In fact, LN has been already employed for the realisation of pumping micro-systems [20,21,22], particles and fluid manipulation devices [23,24,25,26,27] and optical circuits [28]. Some preliminary studies on laser ablation of lithium niobate crystals were investigated to get periodic arrays [29,30], and, recently, even engraving microfluidic channels directly on lithium niobate were consequently reported in the literature by dicing [31,32] and by laser ablation [33,34,35]. In this paper, both laser ablation and dicing were investigated to engrave microfluidic channels on the surface of lithium niobate with the specific aim of defining the best micromachining conditions to reduce the material roughness within the microfluidic channels in order to host integrated optical waveguides facing their lateral walls.

A detailed analysis of the microfluidic channel properties by comparing the results from the laser ablation and the mechanical dicing is therefore reported In microfluidic channels with width and a depth of several tens of micrometres and a length up to few centimetres, respectively. In addition, passive droplet generators realised by both of these techniques are presented, thus proving the actual feasibility of microfluidic devices directly engraved on lithium niobate as well. This work definitively demonstrates the feasibility of integrating efficient droplet microfluidics circuitry in lithium niobate: thanks to its numerous properties (acoustic, electro-optic, photorefractive, pyroelectric), the employment of this material paves the way to original monolithic opto-microfluidic devices fully integrated into the same substrate, not otherwise possible in other standard materials such as glass or silicon in the wide spectral range of light wavelengths starting from 350 nm up to 3 μm, where lithium niobate has an excellent optical transmittivity (better than 75%).

## 2. Experimental

### 2.1. Microfluidic Circuit Realization and Characterization

**Figure 1:**
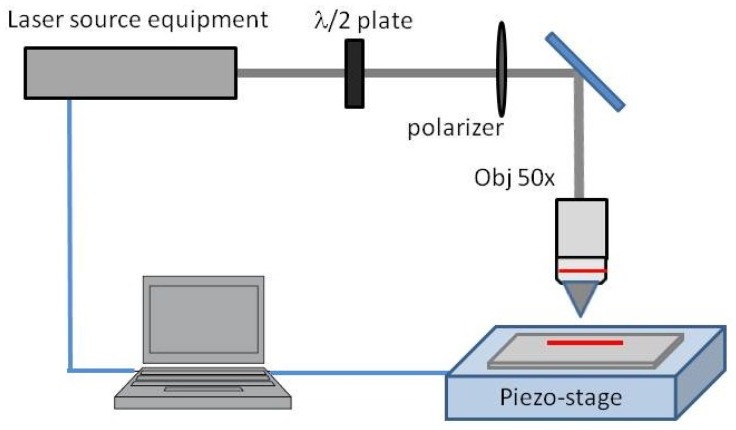
Schemes of the laser ablation set-up used for the material micromachining.

A Ti:Sapphire femtosecond laser with an operating wavelength of 800 nm, a pulse repetition rate of 1 kHz and a pulse duration of 120 fs were focused on the surface of the samples by a 50× ultralong working distance microscope objective (NA = 0.55): a sketch of the experimental apparatus is shown in Figure 1.

The LN crystal was placed on a three-dimensional translational stage controlled by computer driven step motors able to move the sample in all directions with a resolution of a few tens of nanometers. All of the realised structures were obtained scanning the surface with the focused beam with a horizontal step of 10 μm and a vertical step of 15 μm, with grooves being realised by scanning the sample along the main direction of the channels. Samples cut from polished lithium niobate *x*-, *y*- and *z*-cut commercial wafers (Crystal Technology Inc., Palo Alto, CA, USA) were employed to perform the ablation tests, results on *y*-cut samples are here reported more in details as representatives of the micromachining process.

U-shaped grooves were realised on the very edge of the samples in order to measure both the bottom and the lateral walls of the channels. This choice enabled the characterization of the surfaces by means of a Nikon Eclipse Ti-E optical microscope (Nikon Corporation, Melville, NY, USA) and a Veeco CP-II atomic force microscope (AFM, Camarillo, CA, USA). Finally, the scanning electron microscope (eSEM-FEI quanta 200 equipped with an EDAX unit) was used to characterize the surface morphology.

The U-grooves were 600 μm wide along the edge of the samples, extending 150 μm in the direction parallel to the surface and with a depth of 150 μm. Several grooves were engraved varying the laser pulse energy (*E*) from 1 μJ up to 50 μJ and the scanning speed (*v*) in the range 100 to 1000 μm/s, with a pulse energy incident on the material surface and a beam spot size close to 8 μm (as measured by a beam profiler technique at energy full-width-half-maximum (FWHM) signal as a reference). The laser polarisation was kept parallel to the crystal *z*-axis and kept constant during the irradiation process. The experimental conditions were set-up to an energy fluence ranging between 0.5 J/${\mathrm{cm}}^{2}$ and 10 J/${\mathrm{cm}}^{2}$, respectively, above the damage threshold observed in literature (around 0.3 J/${\mathrm{cm}}^{2}$, [34]. Finally, the horizontal/vertical steps in the laser scan were chosen in order to allow for the overlap of two subsequent laser scans but minimizing the final surface roughness.

**Figure 2:**
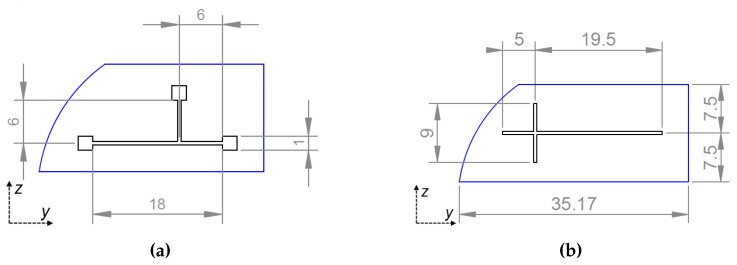
Schemes of the samples engraved with the laser ablation technique (**a**) and the dicing saw (**b**) from +*x* view. Quotes are expressed in millimetres. Curved edges are the round shape of the commercial wafer from which samples were cut.

A T-junction droplet generator configuration was realised by laser ablation technique, with a main channel 18 mm long and a 6 mm long perpendicular channel injecting the dispersed phase (Figure 2a). The versatility of the technique allowed to also engrave three rectangular reservoirs with a side length of 1 mm at the beginning and the end of the channels. The time needed for the whole channel fabrication process was less than three hours. A disco dad 321 precision saw (Disco Corporation, Tokyo, Japan) was used for the mechanical micromachining of the LN samples, with a polymeric blade, coated with diamond particles (blade diameter of 56 mm and nominal thickness of 200 μm) at a rotating speed of 10,000 rpm and a cutting speed of 0.2 mm/s. A constant and high flow of water was employed to keep the sample and the blade at a low temperature during the process and get rid of crystal residuals.

Test channels with a width of 200 μm and a depth of 100 μm, were engraved on samples cut from polished LN commercial wafers by (Crystal Technology Inc.). In order to make both the bottom and the lateral surfaces of the channel reachable by the tip of the atomic force microscope, the samples were then polished from one side until one of the vertical walls of the channel was erased. A cross-junction geometry was implemented on *x*-cut crystals, with two perpendicular crossing channels (200 μm wide and 100 μm thick, respectively) in order to achieve a droplet generator. The dispersed phase was fluxed from one of the perpendicular branches, whereas the opposite one was sealed after being filled with the continuous phase in order to simulate a T-junction configuration. A few minutes are enough for engraving a single cross-junction with dimensions as depicted in Figure 2b.

### 2.2. Microfluidic Characterization Set-Up

The microfluidic channels engraved on the surface of the LN crystals were closed with a silica cover. The cover was attached to the lithium niobate crystal by employing an UV curing Norland Optical Adhesive (NOA68 by Thorlabs Newton, NJ, USA). Fluids were injected into the device through polyethylene tubing. A 100 μM octadecyltrichlorosilane (OTS) solution in toluene was fluxed through the channels in order to make them hydrophobic and allow efficient droplet generation. Two independent automated syringe pumps PHD 2000, (Harvard Apparatus Co., Millis, MA, USA), were used to inject the fluids inside the microfluidic channels through flexible polyethylene tubing in a range from few μL/min up to several tens of μL/min. The injected fluids were hexadecane (Sigma Aldrich, Saint Louis, MO, USA) as the continuous phase and distilled water as the dispersed phase.

Droplet flux monitoring was therefore performed by a fast imaging system, with recording of image sequences with a fast camera connected to a Nikon Eclipse Ti-E microscope (Nikon Corporation, Melville, NY, USA). The microscope was endowed with a Nikon plan 4×/0.10 objective with a 30 mm working distance.

## 3. Results and Discussion

**Figure 3:**
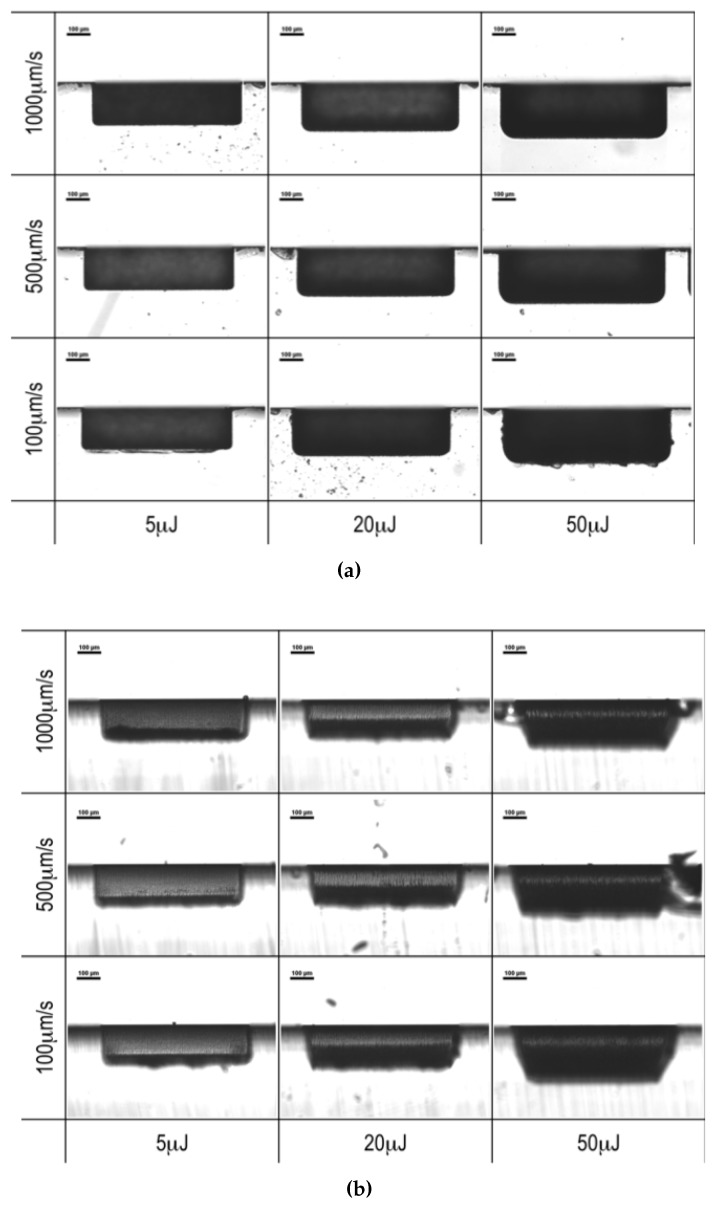
Micrographs of (**a**) the upper edges (+*x* face) and (**b**) the lateral walls (−*y* face) of the test channels engraved by laser ablation on an *x*-cut lithium niobate (LN) wafer. The images are ordered according to the pulse energy and the scanning speed of the laser beam along the horizontal and vertical axes, respectively.

The nominal depth of all of the structures given by laser ablation was set to 150 μm; nevertheless, their actual thickness depends on the pulse energy and the scanning speed employed. The systematic analysis on the role of the laser ablation parameters demonstrated that exceeding the pulse energy above the threshold, as well as the exploitation of a too low scan velocity, can lead to rough edges and scratches or very opaque lateral walls (see Figure 3). On the other hand, energies lower than 5 μJ proved to be unable to remove the material from the surface.

Inside all of the engraved structures, dark residuals affecting the crystal transparency were observed. Sonication lasting several hours in different solvents like ethanol, acetone and acid and basic solutions (${\mathrm{HCl}}_{\left(\mathrm{aq}\right)}$, ${\mathrm{NaOH}}_{\left(\mathrm{aq}\right)}$) were proved to be ineffective in removing these defects. The only way to get rid of darker zones was a bath in a solution containing hydrofluoric acid. In particular, an ${\mathrm{HNO}}_{3}$:HF (3:1 vol.) aqueous solution was used for the etching process.

**Figure 4:**
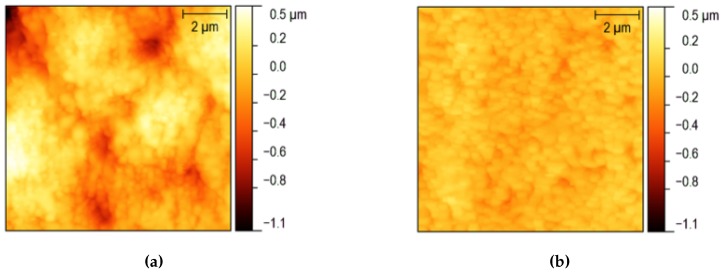
AFM images of the test samples with the lowest roughness obtained by laser ablation. The process parameters are *E* = 5 μJ and *v* = 500 μm/s, and the images refer to the lateral wall (**a**) and the channel bottom (**b**). The colour scale is the same for both of the images in order to allow for a better comparison.

**Figure 5:**
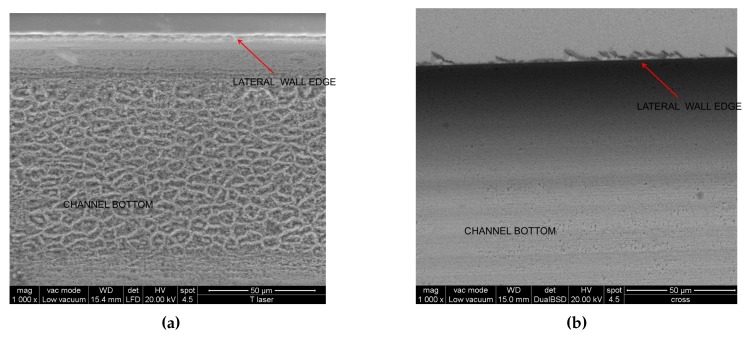
SEM images taken by eSEM-FEI quanta 200 equipped with an EDAX unit with a magnification of ×1000. SEM images of the test samples with the lowest roughness obtained by laser ablation (**a**) and dicing technique (**b**); the top view of a channel engraved by laser ablation at *E* = 5 μJ and *v* = 500 μm/s. SEM images of the test samples with the lowest roughness obtained by laser ablation; the top view of a channel engraved by the dicing technique.

In order to investigate the morphology and measure the average roughness of the channel walls, an accurate Atomic Force Microscopy (AFM) analysis was performed on all the samples (Example in Figure 4), whilst some representatives were analysed by the Scanning Electron Microscope (SEM) technique as well (Example in Figure 5).

**Figure 6:**
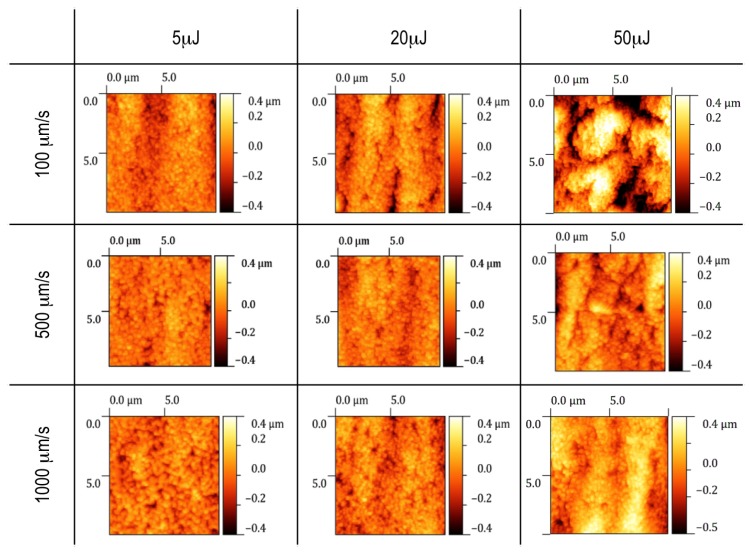
Examples of atomic force microscope (AFM) measurements of 10 × 10 μm^2^ areas on the lateral walls of the microfluidic test channels engraved by laser ablation at different pulse energies and scanning speeds. The colour scale is the same for all of the images in order to highlight the dependence of the surface morphology on the process parameters.

For each test channel, engraved with a different pulse energy *E* and scanning speed *v*, the topography of both the bottom surface and the lateral walls were recorded by means of an AFM (Figure 6).

The average roughness $R_{q}$ of each surface was calculated as the mean value of those obtained on a series of more than five sampled 10 × 10 μm^2^ areas.

**Figure 7:**
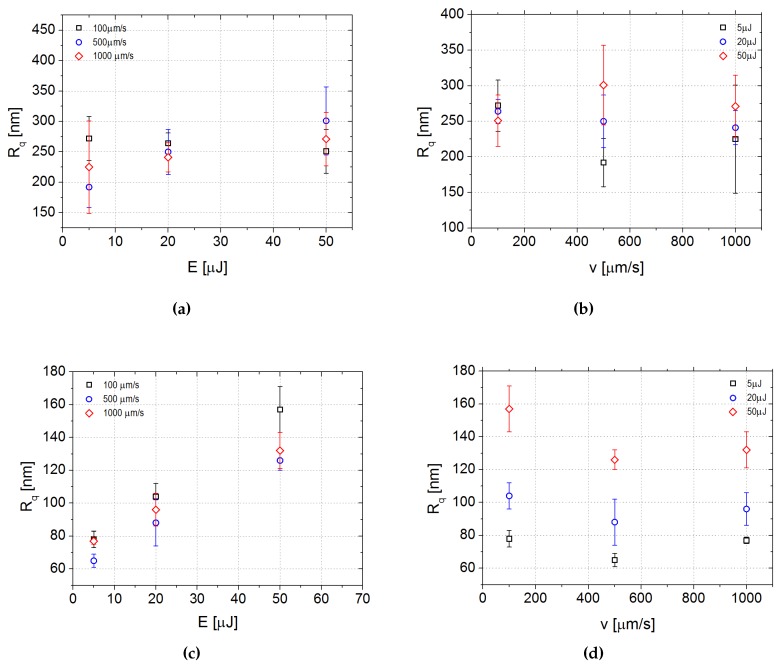
Dependence of the roughness of the channels on the process parameters for the laser ablated samples: (**a**,**b**) $R_{q}$ of the bottom surface of the channels as a function of the pulse energy and the scanning speed, respectively; (**c**,**d**) $R_{q}$ of the lateral surface of the channels as a function of the pulse energy and the scanning speed, respectively.

The bottom walls of the channels have a quite similar morphology though the different pulse energy and scanning speed employed during the ablation process. In all cases, the surface presents small bulges covering bigger rounded hills (see Figure 4a as an example). As a consequence, no clear trend of $R_{q}$ was observed as a function of *E* and *v* (Figure 7a,b).

On the contrary, a clear dependence on the process parameters can be observed on the morphology of the lateral walls. In Figure 6, a few images of 10 × 10 μm^2^ areas are presented according to the pulse energy and the scanning speed employed in the ablation process.

Small bulges at the lowest energies are replaced at the highest energies by deeper cracks aligned with the direction of the laser beam, especially at the lowest scanning speeds, as shown in Figure 6. The morphology change leads to a trend in the average roughness. Figure 7c,d reports $R_{q}$ as a function of the pulse energy and the scanning speed, respectively. $R_{q}$ has a strong dependence on the pulse energy: the higher is the energy above the threshold, the higher is the average roughness of the surfaces.

On the other hand, the roughness has a smoother dependence on the scanning speed and seems to present a minimum at an intermediate value of the velocity.

The lower average roughness ($R_{q}$) was obtained with a pulse energy of 5 μJ and a scanning speed of 500 μm/s, and it was found to be 192 ± 34 nm and 65 ± 4 nm for the bottom and the lateral surface, respectively. Two images of the surfaces realised with these process parameters are reported in Figure 4 as an example. In this case, the SEM measures (Figure 5a ) showed that the roughness in randomly distributed, without any memory effect to the scanning protocols but presenting a sort of patched style in the 5 to 8 μm scale respectively. A SEM image is shown in Figure 5a, where the top view of the engraved microfluidic channel is presented with a magnification of ×1000.

**Figure 8:**
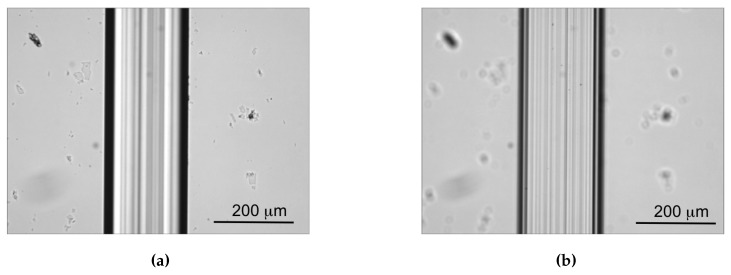
Micrographs of the upper edges (**a**) and the bottom surface (**b**) of the microfluidic channels obtained by mechanical micromachining with the dicing saw.

These values of roughness are aligned with the results obtained on silica by similar approaches [19,36]. The microfluidic channels engraved with the disco dad 321 dicing saw were found to have very sharp edges and transparent walls (Figure 8). A SEM image is shown in Figure 5b, where the top view of the engraved microfluidic channel is presented with a magnification of ×1000 (i.e., the same as that reported in Figure 5a for comparison). The bottom roughness presents a sort of texturing parallel to the channel length derived from the effect of the blade scan, but the overall roughness is lower than that observed in the laser ablated channels. Some imperfections can be seen at the corner of the lateral wall edge that, however, can be minimised by exploiting the optimised scan speed.

Moreover, after a standard cleaning process by sonication in baths of distilled water, isopropanol and finally acetone, no residuals were observed on the surfaces of the channels.

**Figure 9:**
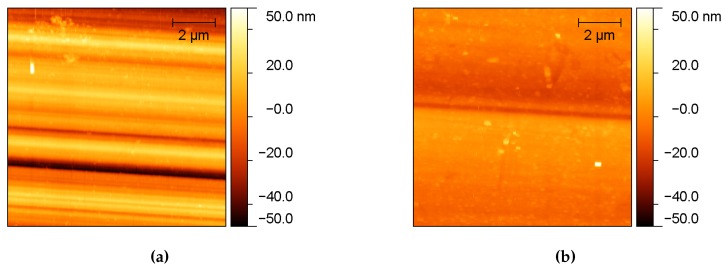
10 × 10 μm^2^ AFM images of the bottom surface (**a**) and the lateral surface (**b**) of the test channels realised by mechanical micromachining with the dicing saw. The colour scale is the same for both of the images to allow for a better comparison.

AFM analyses in the same conditions employed for the laser ablated channels were performed in a systematic way and are reported here for comparison. As already mentioned, a lower roughness was measured in this case, an average $R_{q}$ was measured to be 23 ± 7 nm for the bottom surface and 8.5 ± 0.9 nm for the lateral wall of the channels (Figure 9). These values of roughness are compatible with an optical grade quality of the surface, even for wavelengths in the visible range, thus paving the path for applications in the field of optofluidics, such as the coupling between microfluidic channels and optical waveguides.

### Droplet Generator Performances and Droplet Length Scaling Relation

The final microfluidic performances of the droplet generators made by laser ablation and dicing were tested and compared in terms of quality, reproducibility and rate of the generated droplets, respectively, in the the squeezing regime (i.e., when the tip of the dispersed phase fills completely the main channel before the droplets breakup takes place). This regime is, in fact, verified at low values of the capillary number [37]:

(1) $$\mathrm{Ca}=\frac{\mu_{c}Q_{c}}{\sigma w_{c}h}<0.015,$$

where $\mu_{c}$ is the dynamic viscosity of the continuous phase, $Q_{c}$ is the continuous phase flow rate, σ the interfacial tension between the two fluids, $w_{c}$ and *h* the main channel width and height, respectively. In particular, several tests were made on water droplets dispersed in oil, as a perspective to several biological applications, varying the flow rates $Q_{d}$ and $Q_{c}$ of the dispersed (DI water) and the continuous phase (hexadecane) in a wide range. For each couple of flow rates, statistical analysis was carried out recording lengths of more than 100 droplets, estimating the relative size distribution and standard deviation ($\sigma_{L}$). The relative dispersion $\sigma_{L}\left(\%\right)=100\cdot \sigma_{L}/L$ of the length distribution was considered as an indication of the performance level of the device in generating droplets at a fixed volume. In addition, the length of the droplets (*L*) rescaled on the width of the channel (*w*) was studied as a function of the flow rate ratio $\phi =Q_{d}/Q_{c}$. As proposed for the first time by Garstecki et al. [38] and confirmed by several authors later, the rescaled length L/w of the droplets produced in a T-junction has a linear dependence on ϕ in the squeezing regime:

(2) $$\frac{L}{w}=\alpha +\beta \phi ,$$

where the parameters α and β are two fitting parameters depending mainly on the junction geometry [39].

**Figure 10:**
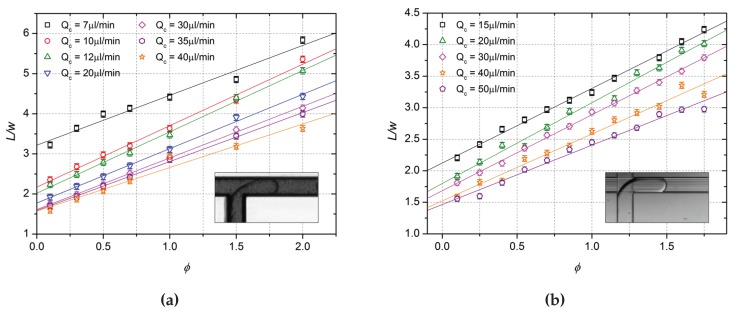
Dependence of the rescaled length L/w on the flow rate ratio at different values of the continuous phase flow rate $Q_{c}$ with the relative linear fit. the data obtained with the laser ablated T-junction (**a**); the data from the cross-junction engraved with the dicing saw (**b**).

The length distribution of the droplets produced with the T-junction by laser ablation was measured varying the dispersed phase flow rate in the range $Q_{d}$ = {0.1, 0.3, 0.5, 0.7, 1.0, 1.5, 2.0}$\cdot Q_{c}$ for each value of the continuous phase flow rate in $Q_{c}$ = {7, 10, 12, 20, 30, 35, 40} μL/min, respectively. The relative dispersion $\sigma_{L}\left(\%\right)$ was found to be lower than 3% in all cases, indicating a high repeatability, perfectly aligned with the best results reported in literature for T-junctions obtained in different substrates [40,41,42,43]. In particular, Equation (2) was verified for all of the different imposed flow rates of the continuous phase as depicted on Figure 10a, where it is possible to observe the good agreement between the experimental data and the linear fits (analogue performances were obtained for the cross-junctions engraved by means of the dicing saw, employed as a T-junction droplet generator. The length relative dispersion was found to be always below 5%, and, on average, lower than 2%. The tested flow rates were $Q_{d}$ = {0.1, 0.25, 0.4, 0.55, 0.7, 0.85, 1.15, 1.3, 1.45, 1.6, 1.75}$\cdot Q_{c}$ and $Q_{c}$ = {15, 20, 30, 40, 50} μL/min. Even in this case, the linearity between the rescaled droplet length and the flow rate ratio ϕ was verified in the whole range of the investigated flow rates as reported on Figure 10b.

## 4. Conclusions

**Figure 11:**
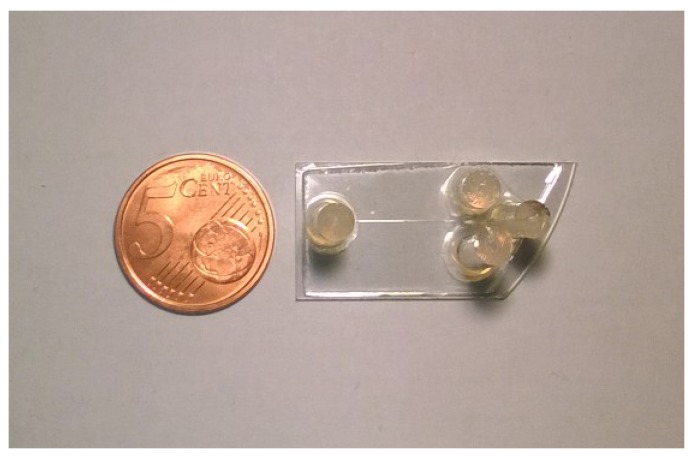
Photo of one of the tested microfluidic devices whose channels were realised by means of the dicing saw.

This work demonstrates that microfluidics channels with the typical dimensions required in microfluidics applications can be achieved in lithium niobate crystals by micromachining techniques (example of the final device in Figure 11). Depending on the micromachining parameters, the roughness of the channel lateral wall and bottom can vary on an order of magnitude and can allow for the integration of optical waveguides. In particular, both the laser ablation and the mechanical micromachining by means of a dicing saw were proven to be effective in engraving the LN crystals, providing a high quality of the obtained channel surfaces.

The great advantage of laser ablation relies also in the fact that the microfluidic design is easier, channels can be shrunk or enlarged, and their depth can in fact be tailored with no added complexity.

The mechanical micromachining guarantees shorter times of production and the extremely low roughness of the channel surfaces is suitable for future optofluidic applications [44] (see Table 1 for a summary of the comparison between the key parameters of the two micromachining techniques). Although the laser ablation technique is more flexible because it allows for designing microfluidic circuits with any desired two-dimensional geometry, the final roughness is higher than that achieved by dicing, provided that special blades that are used are those exploited in this work. For integrated opto-fluidic perspectives, therefore, the best solution is a combination of the two, where high quality dicing is needed when integrated optical waveguides are coupled by facing the microfluidic lateral wall, respectively.

Furthermore, passive droplet generators realised by micromachining a lithium niobate crystal were shown to ensure a steady production of monodispersed water droplets in oil with a very tight distribution of their volume (relative dispersion lower than 3% in the case of the laser ablated T-junction, lower than 5% for the diced cross-junction), perfectly aligned to the best microfluidic devices realised with other materials.

In addition, the verified linear relation between the rescaled droplet length L/w and the flow rate ratio ϕ (Equation (2)) allows for predicting with accuracy the volume of the droplets according to the imposed fluxes. As a final consideration, the most promising perspective of this work is the joint exploitation of both the laser ablation technique and the mechanical micromachining one. As a matter of fact, the first one could be employed to realise more complex microfluidic circuits, whereas the high quality of the surfaces obtained with the second one would allow to integrate optical stages to the microfluidic channels. In this way, it will be possible to fabricate microfluidic devices with geometries as complex as those made by polymeric materials, and coupling on the very same substrate integrated optical stages thanks to the well-known optical properties of lithium niobate, which, among others, is already widely used for the realisation of waveguides and diffraction gratings.

## Figures and Tables

**Table 1:** Summary of the key parameters for the droplet generators realised with the two micromachining techniques presented in this paper.

Technique	Fabrication Time	$R_{q}$[nm]		Droplet Length Dispersion
		Bottom	Side	
Laser Ablation	3h	192 ± 34	65 ± 4	<3%
Dicing Saw	Few Minutes	23 ± 7	8.5 ± 0.9	<5%
